# Editorial: The Mental Lexicon, Blueprint of the Dictionaries of Tomorrow: Cognitive Aspects of the Lexicon

**DOI:** 10.3389/frai.2022.945705

**Published:** 2022-07-18

**Authors:** Michael Zock, Simon De Deyne, Massimo Stella, Vito Pirrelli

**Affiliations:** ^1^CNRS, Aix Marseille Université, Group TALEP, Marseille, France; ^2^School of Psychological Sciences, The University of Melbourne, Parkville, VIC, Australia; ^3^CogNosco Lab, Department of Computer Science, University of Exeter, Exeter, United Kingdom; ^4^Institute for Computational Linguistics, National Research Council (CNR), Pisa, Italy

**Keywords:** mental lexicon, words, dictionary, brain, cognition, neural networks, artificial intelligence

Words are the building blocks of language. OK, but what are words? Are they universal? Where and how are they stored, represented and organized in the human brain, i.e., mental lexicon (henceforth, ML)? These are important questions, and their answers are far from simple. Hence our motivation for this Research Topic.

Concerning *words*: Let us consider them as textual items delimited by a space, or as headwords (lexical entries) listed in a dictionary. They both are words, but they are not the same. This is even more true when we consider words in the human brain, or the ML.

Words in dictionaries are building blocks, i.e., raw material, appearing there in a standardized form (lemma), out of context, and devoid of morphosyntactic information revealing tense, number, or the word's actual role (agent, object, instrument). Unlike the ML, which is limited in size and highly personal, dictionaries contain the lexical competency of a very large community. Having been built by a team of experts, they are more precise and larger than anyone's personal lexicon. Yet, being disconnected from the real world (personal experience, feelings, knowledge), they can tell us only part of the story. Take any entry, say “dog.” Its definition is only a poor substitute for the pet a child has in mind when referring to Ginger, its beloved animal.

There are other problems like *multi-word expressions* (idioms, phrasal verbs, collocations), or the impact of the *writing system*. Having grown up in a culture that uses an alphabet, you will have no trouble to recognize words, even in a foreign language, as long as the writing is the same (sound to grapheme mapping). The situation is quite different though for a language like Chinese which uses a logographic-, or, more precisely, a morpho-syllabic writing system. By comparing a given message in Chinese and English—我的泰勒很有 (Wo de tàilēi hěn you qián) vs. “My tailor is rich”—, you will realize that it is not easy to recognize words in Chinese. Indeed, out of seven characters only five are words: 我,的,泰勒,很,有.

The situation is even more complex when we compare lexical entries and words in the ML. In dictionaries, they are holistic entities, i.e., objects (word forms), available at any time. In the brain they are decomposed, their elements being distributed. Yet, if any of them lacks energy, the word's full form may not be accessible anymore. Words in the brain are only virtual entities, i.e., abstract patterns that become “realized” over time: **1**° *concept* : 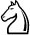 ⇒ **2**° *abstract word* : horse [sing, N] ⇒ **3**° *concrete form* = h rc s/horse. This is why psychologists prefer to use the term “activation” to refer what lexicographers call the “look up” of a word. Some see words as the result produced by a word factory (brain), while others consider them as existing entities, stored in a database. One last point: the human mind does not make any distinction between the lexicon, the thesaurus, topic maps, named entities, episodic- or encyclopedic knowledge. They all are integrated into a single resource, called upon depending on the momentary cognitive state or knowledge need.

A lot of work has been devoted to the ML, but given our current knowledge state it is hardly more than a fascinating metaphor. For example, we do not have a clear idea concerning its topology, nor do we know how to build this map. This is what motivated our Research Topic, and here are some of its goals: (a) clarify the notion of mental lexicon; (b) consider its value for building navigational tools to support word finding (c) conceive a strategy to build the map, (d) draw attention to work in other disciplines to foster cross-fertilization.

This Research Topic contains seven papers. The first two focus on the notion of the ML.

In the manifesto Zock tries to answer the question raised in the title. Being interested in the development of brain-compatible software, he offers some guidelines for the development of a tool to help authors to overcome the tip-of-the-tongue problem. Observing that people resort to different resources (dictionary, thesaurus, encyclopedia) depending on their knowledge state, which is variable and unpredictable, he suggests integrating all of them into a single resource.

Taking inspiration from Quantum Physics, Libben tries to give more substance to the notion of the ML. To this end he introduces two innovative principles of lexical organization: *morphological transcendence* (key ⇒ keyboard ⇒ turnkey) and *lexical superstates* ([wallpaper = noun] ⇒ [to wallpaper = verb])

McCrae et al. address the question of how to determine the number of senses words have. They tackle this problem by comparing the strengths and weaknesses of formal, cognitive, distributional and intercultural approaches. Their work provides a detailed case study for “fish” and “wing.” As there are substantial variations concerning the number of senses identified with each approach, the authors show how they might be unified.

Aguirre-Celis and Miikkulainen explored the changes of the conceptual representation of a word. If a given word occurred in different contexts, its meaning changes would be signaled *via* different brain-level representations. The word's meaning may change dynamically in the ML, the actual meaning depending not only on the concept's inherent features but also on contextual information.

Going beyond word length and frequency effects, Hofmann et al. could show to what extent semantic and syntactic factors affect word access in the ML. By predicting single fixation, gaze and total viewing duration for reading, the authors showed that n-gram and recurrent neural network representations of words allow for better performance than topic models or cloze probability.

The ML is understood to “contain” not only morphologically simple words, but also inflected and derived forms, compounds, light verb construction, collocations, idioms, social routine clichés and pre-compiled routinized chunks maximizing processing opportunities. Yang et al. show that a simple segmentation model inspired by Zipf's *principle of least effort* can simulate the acquisition of such assorted lexical materials, and account for their use by readers of a connected text.

In the final paper Jacobs and Kinder discuss the potential and limitations of distributed semantic models (DSM) to account for the gain of knowledge (apperceptive mass) to enrich the ML, *via* reading books. The results show that performance increases with age, and that DSMs, while promising, are nevertheless still far from perfect.

While the here-presented papers look promising, more work is needed. Describing the structure and functioning of the ML is bound to keep the research community busy for quite some time.

## Author Contributions

All authors listed have made a substantial, direct, and intellectual contribution to the work and approved it for publication.

## Conflict of Interest

The authors declare that the research was conducted in the absence of any commercial or financial relationships that could be construed as a potential conflict of interest.

## Publisher's Note

All claims expressed in this article are solely those of the authors and do not necessarily represent those of their affiliated organizations, or those of the publisher, the editors and the reviewers. Any product that may be evaluated in this article, or claim that may be made by its manufacturer, is not guaranteed or endorsed by the publisher.

